# Hernia of the Bladder

**Published:** 1909-10-02

**Authors:** 


### Surgery.

### HERNIA OF THE BLADDER.

Of all the curious and unexpected structures met with during an operation for the radical cure of an inguinal hernia, the bladder most frequently passes unobserved—sometimes with disastrous consequences.

This viscus may complicate a hernia in a variety of ways. In those operations for radical cure which necessitate that the inguinal canal be widely opened and the neck of the sack much manipulated, it sometimes happens that excessive traction on the peritoneum will pull down a portion of the lateral wall of the bladder, which then comes to lie at the inner pillar of the ring and may perhaps be included in the embrace of the ligature and cut away.

This variety of hernia is therefore in the nature of an artefact, made by the surgeon; but it is obvious that if the hernia be a recurrent one, bladder may be pulled down by the peritoneum in its descent to form the new sac; so that a veritable hernia of the bladder may exist at the time of operation. If much bladder has descended it may be recognised as a bulging into the upper and inner wall of the sac, but when less obvious the risk of its unsuspected inclusion in the ligature is again considerable.

A hernia of the bladder of this type has occasionally been of such size as to be recognisable clinically. It is usually found as a complication of extensive direct herniæ in old men with weak belly walls, and has, or may have, well-defined clinical features of which the "micture à deux temps" is perhaps the best marked. Pressure on the sac may give rise to an urgent desire for micturition. The passage of a strongly-curved metal catheter will complete the diagnosis of such a hernia if the point can be made to pass into the adventitious pouch.

The cases so far referred to are those in which the presence of the bladder is an incident, as it were, in an inguinal hernia of otherwise ordinary character.

Of greater interest are the rare cases, all of congenital origin, in which the herniation of bladder is the primary factor and a peritoneal sac, if present, has followed secondarily, forming a pouch upon the upper and outer aspect of the prolapsed bladder. The peritoneal sac may or may not be larger than the portion of bladder implicated, for it is obvious that once the peritoneum has commenced to descend, the pressure of the intestines will further its descent, while the bladder has no such impetus behind it.

As already indicated, bladder may descend alone, unaccompanied by peritoneum, and may form a quite considerable pouch in the scrotum, as in a case recorded by Corner in a young infant, which was further remarkable for the fact that the pouch was the seat of tuberculosis. This form of hernia is, however, excessively rare, the commonest variety being that in which bladder and peritoneum co-exist in equal quantity, as it were, although none of them is at all frequent.

### Ætiology.

The ætiology of these herniæ is just as mysterious as that of the sliding herniæ of the cæcum and the vermiform appendix with which it will be apparent that they have much in common.

Possibly, the vagaries of that mysterious and probably much maligned structure, the *gubernaculum testis*, may be cited as a possible explanation of their formation. In any case, until further information upon the subject is forthcoming, the explanation seems a feasible one.

### Diagnosis.

Probably no injustice will be done to anyone if it be affirmed that not one case in ten is recognised before operation. Even when cases come to operation, it sometimes happens that the passage of blood in the urine or the escape of urine through the wound are the first indications that the bladder has entered into the field of operation.

The most important thing to bear in mind is that when in the course of a radical cure of hernia there is difficulty in defining the sac, or the case in other ways appears abnormal, the presence of bladder or some other abnormal constituent should be suspected, and extreme care taken to avoid wounding it. If bladder is present, careful dissection through the layer of fat with which it is always covered will reveal its easily-recognised reddish-brown muscular fibres and give a clue to the nature of the case.

The cæcum—uncovered by peritoneum, as it is sometimes in sliding hernia of the cæcum—might be confounded with it in right-sided cases; but the muscle fibres of the bladder are of a much browner tint, and furthermore the bladder is always at the inner aspect of any peritoneal sac present, while the cæcum would be to the outer side.

### Treatment.

The question of treatment depends upon whether the abnormal state of affairs has been recognised before or after the bladder has been cut into.

If the condition is recognised early, then the bladder must be returned into the abdomen and the peritoneal sac with the inguinal canal treated in the usual way. When the sac is very small it may be found impossible to separate it from the bladder for the purpose of ligaturing it. In that case the neck of the sac may be closed by a purse-string suture, which at the outer part takes up the superficial fibres of the bladder.

If the bladder has been wounded, the wound should be closed with two layers of fine catgut sutures—for if non-absorbable suture material be used, it has an unpleasant custom of working through the mucous membrane into the cavity of the bladder and forming the nucleus of a calculus. The external wound should not entirely be closed, a small tube being left in for forty-eight hours in case the sutures in the bladder wall are not watertight, or rather urine tight.

In cases recognised early and dealt with on these lines convalescence should be free from unhappy complications and sequelæ.

